# A Compact High-Speed Image-Based Method for Measuring the Longitudinal Motion of Living Tissues

**DOI:** 10.3390/s20164573

**Published:** 2020-08-14

**Authors:** Ruilin Yang, Heqin Liao, Weng Ma, Jinhua Li, Shuxin Wang

**Affiliations:** 1 Key Laboratory for Mechanism Theory and Equipment Design of Ministry of Education, School of Mechanical Engineering, Tianjin University, Tianjin 300350, China; ruilinyang@tju.edu.cn (R.Y.); tjumemw@tju.edu.cn (W.M.); lijinhua@tju.edu.cn (J.L.); 2 National Ocean Technology Center, Tianjin 300112, China; liao_heqin@126.com

**Keywords:** motion of living tissues, active motion compensation, image-based method, simple structure, animal experiment

## Abstract

Intraoperative imaging of living tissue at the cell level by endomicroscopy might help surgeons optimize surgical procedures and provide individualized treatments. However, the resolution of the microscopic image is limited by the motion of living tissue caused by heartbeat and respiration. An active motion compensation (AMC) strategy has been recognized as an effective way to reduce, or even eliminate, the influence of tissue movement for intravital fluorescence microscopy (IVM). To realize the AMC system, a high-speed sensor for measuring the motion of tissues is needed. At present, state-of-the-art commercialized displacement sensors are not suitable to apply in minimally invasive imaging instruments to measure the motion of living tissues because of the size problem, range of measurement or the update rate. In this study, a compact high-speed image-based method for measuring the longitudinal motion of living tissues is proposed. The complexity of the proposed method is the same as that of the traditional wide-field fluorescent microscopy (WFFM) system, which makes it easy to be miniaturized and integrated into a minimally invasive imaging instrument. Experimental results reveal that the maximum indication error, range of measurement and the sensitivity of the laboratory-built experimental prototype is 150 μm, 6 mm and −211.46 ${\mathrm{mm}}^{-1}$ respectively. Experimental results indicate that the proposed optical method is expected to be used in minimally invasive imaging instruments to build an AMC system.

## 1. Introduction

It is widely recognized that intraoperative diagnosis technologies contribute to minimize surgical damage, preserve normal tissue and reduce the rate of recurrence for tumor resection surgery [1,2,3,4,5]. Optical imaging is characterized by low cost, nonionizing radiation and simple and high resolution [6,7,8], which is promising for design of an intraoperative diagnosis instrument. Despite advances in technology, the motion of living tissues or organs caused by breathing or heartbeat remains a major problem in intraoperative imaging by minimal invasive microscopic imaging devices (e.g., endomicroscopy) for pathologic diagnosis [9,10,11].

In the past decade, it has been validated that active motion compensation (AMC) is an effective way to reduce, or even eliminate, the influence of tissue motion when imaging living tissue in vivo for intravital fluorescence microscopy(IVM) [12,13,14]. AMC eliminates the influence of tissue movement by adding an additional synchronous motion to the imaging system to replicate the motion of tissues and eliminate the relative movement. Thus, to realize an AMC system for eliminating the effect of tissue movement, a high-speed displacement sensor for measuring the motion of living tissues in real time is indispensable.

**Figure 1:**
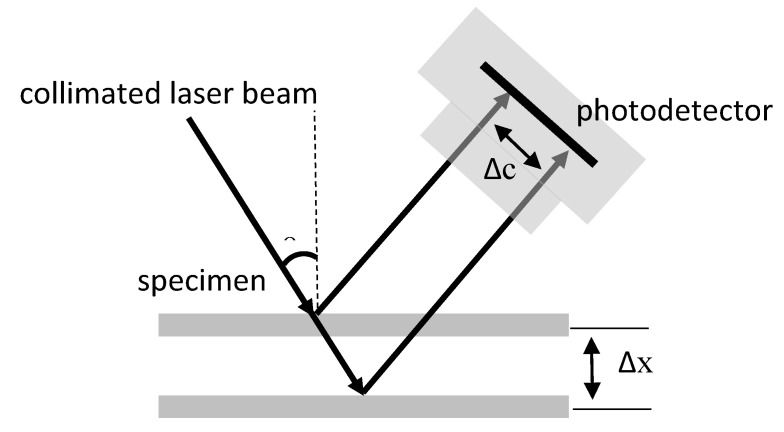
Illustration of optical triangulation method for measuring the displacement of specimen. A well collimated laser beam irradiates the surface of the specimen at an angle, the reflected beam is collected by a photodetector, and thus the displacement of the specimen can be estimated by the position of the imaged laser spot.

Currently, state-of-the-art methods for measuring the motion of living tissues are based on an optical system, such as optical triangulation [15,16,17,18,19,20], high speed optical imaging [12,21], optical coherence tomography (OCT) [22,23] and image correlation computation [14,24]. Optical systems can be further broadly classified as nonimage-based and image-based systems. Among them, the optical triangulation method is one of the most famous nonimage-based methods to measure small displacement of the specimen [15,16,17,18,19], which has a well collimated laser beam to irradiate the surface of the specimen at a certain angle, as shown in Figure 1. The displacement of the specimen (Δx) can be determined by detecting the displacement of the imaged laser beam spot (Δc). The main advantages of the optical triangulation method are high speed and accuracy, based on which a lot of commercialized laser displacement sensors have been designed. The limitation of this method is the complex structure, which makes it not easy to integrate with the front end of a minimally invasive instrument. For the image-based system, the displacement of the living tissue is estimated according to the image captured by the image sensor (e.g., CCD, CMOS) through complex image processing [12,14,21,22,23,24]. The optical imaging system, especially the wide-field image system, has a simpler structure than the optical triangulation system, which makes it easy to be miniatured and integrated into a minimally invasive instrument, but it still has some limitations to measure the displacement of living tissues with image-based methods. One of the major limitations is that it commonly measures the movement of living tissues in the horizontal (X-Y) direction with high-speed optical imaging system [12,21], but it is not easy to measure the movement of living tissues in the longitudinal (Z) direction with a single-camera-based imaging system. Although the OCT method can achieve micron level measurement accuracy for the longitudinal movement of living tissue, the high measuring precision means a small measuring range [22,23]. Recently, a method that estimates 3D motion of living tissues by correlation computation has been proposed [14,24]. However it consumes a huge amount of time, which limits the sampling rate of measurements.

In this paper, a compact high-speed image-based method for measuring the longitudinal motion of living tissues is proposed. We also built an experimental prototype to validate the effectiveness of the proposed method. The subsequent part of this paper is organized as follows. In Section 2, the proposed method and experimental protocols are described. The experimental results are illustrated in Section 3. Discussions and conclusions of this study are given in Section 4 and Section 5, respectively.

## 2. Materials and Methods

### 2.1. System Design and Implementation

**Figure 2:**
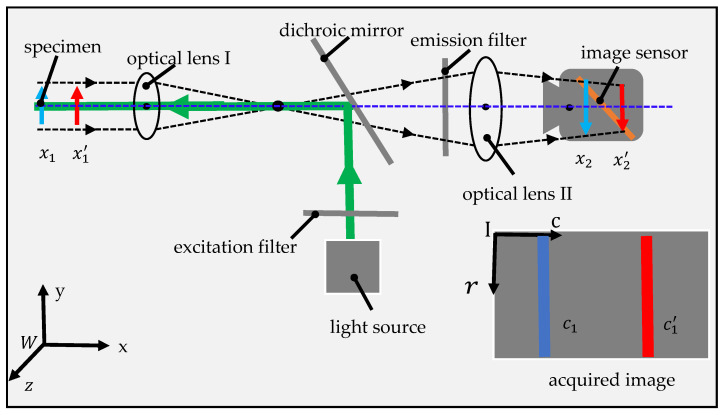
Optical diagram of high-speed image-based system for measuring the motion of living tissues. The system illustrated above has a simple structure, which makes it easy to integrate into the front end of a minimally invasive instrument to measure the motion of living tissues in vivo.

The optical structure of the high-speed image-based motion detection system proposed in this paper is illustrated in Figure 2, which mainly consists of an image sensor, two optical lenses, a dichroic mirror (DM), a light source and excitation and emission filters. The light from light source is reflected by the DM, and then projected onto the specimen after being focused by optical lens I. Fluorescence signals from the specimen are collected by optical lens I, then transmitted through the DM and emission filter, focused by optical lens II, and finally collected by the image sensor.

The above proposed system is similar to traditional wide-field fluorescent microscopy (WFFM) system [25,26,27]. The main difference between them is the direction of the image sensor. In a WFFM system, the plane of the image sensor is parallel with the image plane, and the image sensor can acquire the entire image of the specimen in the field of view (FOV). However, in the proposed system, the image sensor plane is not parallel with the image plane. Only the intersection line can be sensed by the image sensor and only a stripe region of the specimen can be imaged. According to optical imaging theory, the distance between optical lens II and the image plane can be regulated by the distance between optical lens I and the specimen. Suppose that the specimen plane and the corresponding image plane are initially located at $x_{1}$ and $x_{2}$ in the world coordinate system (W−xyz). The image plane will move from $x_{2}$ to $x_{2}^{\prime }$ if the specimen moves from $x_{1}$ to $x_{1}^{\prime }$. At the same time, the intersection line will move from $c_{1}$ to $\;c_{1}^{\prime }$ in the image coordinate system (I−rc). On account of above analysis, we can measure the movement of the specimen by calculating the movement of the stripe in the captured image.

**Figure 3:**
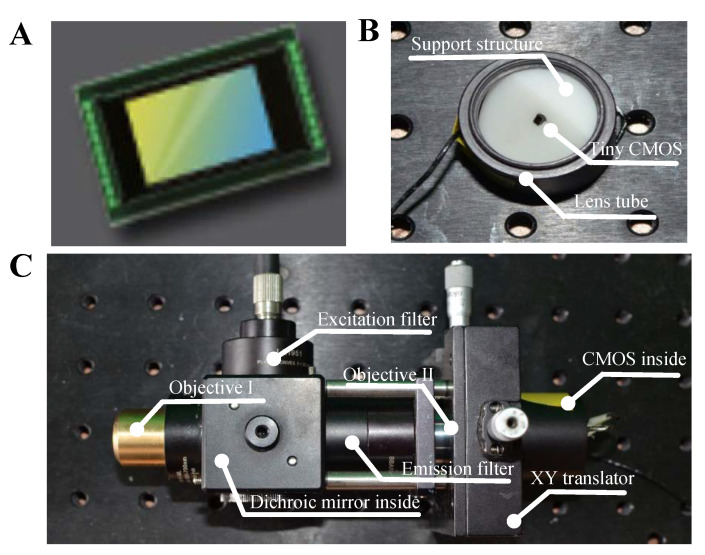
Photograph of the laboratory-built prototype, (**A**) image sensor [31], (**B**) the tube with image sensors mounted and (**C**) the photograph of prototype.

We chose a plan achromat objective with 4X magnification (RMS4X, Thorlabs) and 10X magnification (RMS10X, Thorlabs) as optical lens I and optical lens II, respectively. A laser diode (wavelength: 445 nm, output power: 0–2 W) was used as the illumination source. The dichroic mirror (DM) (reflection band: 415–470 nm, transmission band: 490–720 nm, MD480, Thorlabs), excitation filter (center wavelength:445 nm, FWHM:45 nm, MF445–45, Thorlabs) and emission filter (center wavelength: 510 nm, FWHM: 42 nm) were selected based on the spectral characteristics of the fluorescent agent of proflavine (Sigma-Aldrich), which has a peak excitation wavelength of 445 nm and a peak emission wavelength of 510 nm [28,29,30]. The image sensor in our system (Figure 3A) was a tiny CMOS sensor (1280 × 720 pixels, pixel size: 1.116 μm, image area: 1.44 mm × 0.91 mm, die dimensions: 2.53 mm × 1.53 mm, OH01A10, Omni vision) and the frame rate of the tiny CMOS was 30 frame/s. To make the image sensor plane at an angle of 45-degree with the optical axis, we utilized a 3D printer to make a solid cylinder structure with a 45-degree slope to support and fix the image sensor. Then we put the printed support structure into a lens tube and fixed it by a retaining ring, as shown in Figure 3B. Finally, the lens tube with the image sensor was mounted in an XY translator (ST1XY-S, Thorlabs), which was used to align the image sensor with the optical axis. A photograph of the laboratory-built prototype is shown in Figure 3C.

### 2.2. Mathematical Model of Prototype

**Figure 4:**
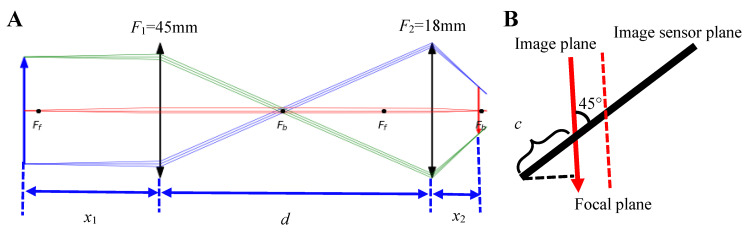
Modeling parameters of optical imaging system. (**A**) The optical system consisting of two objective lenses with an effective focal length (EFL) of 45 mm and 18 mm, respectively. The distance from specimen plane to objective lens I is represented by $x_{1}$, and the two objective lenses are separated by a distance of d. The distance from objective lens II to the image plane is represented by $x_{2}.$ (**B**) The geometric relationship between image plane and image sensor plane.

The quantitative relationship between the motion of the specimen and the formed image plane can be derived from the ray transfer matrix (ABCD matrix) [32,33,34]. Assuming that the focal lengths of optical lens I and optical lens II are $F_{1}$ and $F_{2}$, respectively, the two optical lenses are separated by a distance of d, the specimen is placed at a distance of $x_{1}\;$ to the optical lens I and the image plane is at a distance of $x_{2}$ to the optical lens II, the optical scheme can be simplified as shown in Figure 4A.

If the specimen plane is considered as the input plane and the image plane as the output plane, the ABCD matrix of the overall optical system can be determined by multiplication of all basic optical components, as

(1) $$(\begin{array}{cc} A & B\\ C & D \end{array})=\left(\begin{array}{cc} 1 & x_{2}\\ 0 & 1 \end{array}\right)\left(\begin{array}{cc} 1 & 0\\ -1/F_{2} & 1 \end{array}\right)\left(\begin{array}{cc} 1 & d\\ 0 & 1 \end{array}\right)\left(\begin{array}{cc} 1 & 0\\ -1/F_{1} & 1 \end{array}\right)\left(\begin{array}{cc} 1 & x_{1}\\ 0 & 1 \end{array}\right)$$

The elements of ABCD matrix can be derived as

(2) $$A=1-\frac{x_{2}}{F_{2}}-\frac{d}{F_{1}}+\frac{dx_{2}}{F_{1}F_{2}}-\frac{x_{2}}{F_{1}}$$

(3) $$B=x_{1}+x_{2}+d-\frac{x_{1}d}{F_{1}}-\frac{x_{1}x_{2}}{F_{2}}-\frac{x_{2}d}{F_{2}}-\frac{x_{1}x_{2}}{F_{1}}+\frac{x_{1}x_{2}d}{F_{1}F_{2}}$$

(4) $$C=-\frac{1}{F_{1}}-\frac{1}{F_{2}}+\frac{d}{F_{1}F_{2}}$$

(5) $$D=1-\frac{x_{1}}{F_{1}}-\frac{x_{1}}{F_{2}}-\frac{d}{F_{2}}+\frac{x_{1}d}{F_{1}F_{2}}$$

Since it is necessary for the element B  in the overall ABCD matrix to be zero to achieve image formation, the relationship between $x_{1}$ and $x_{2}$ can be derived as

(6) $$x_{2}=\frac{dx_{1}F_{2}-dF_{1}F_{2}-x_{1}F_{1}F_{2}}{F_{1}F_{2}-x_{1}F_{2}-x_{1}F_{1}-dF_{1}+dx_{1}}$$

When B=0,  the value of element A gives the overall magnification of the image. Therefore:

(7) $$M=A=1-\frac{x_{2}}{F_{2}}-\frac{d}{F_{1}}+\frac{dx_{2}}{F_{1}F_{2}}-\frac{x_{2}}{F_{1}}$$

Supposing that the image sensor is located at the focus of optical lens II, the width of the image area of CMOS is W, the total column number of pixels in the captured image is $N_{c}$ and the column in the image coordinate system is c, according to the geometric relation shown in Figure 4B the relationship between the stripe in the image coordinate system and the position of real image can be expressed as:

(8) $$c=\frac{x_{2}-F_{2}+W*\cos(45{}^{\circ})/2}{\cos(45{}^{\circ})}\frac{N_{c}}{W}$$

### 2.3. Image Preprocessing

**Figure 5:**
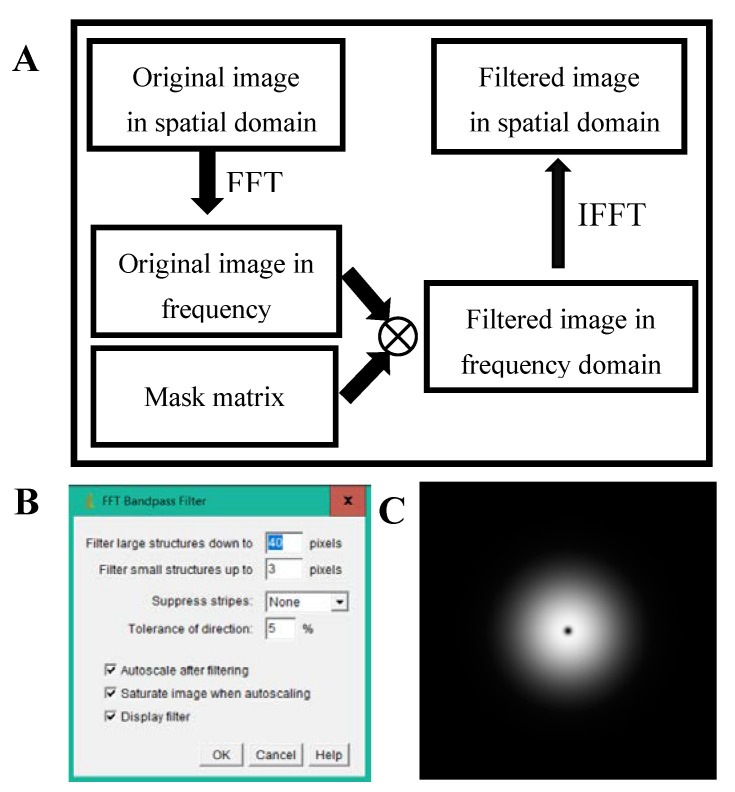
(**A**) The generalization process of bandpass filter based on fast Fourier transformation (FFT). (**B**) The parameters of the mask matrix with Image J software. (**C**) The mask matrix of the bandpass filter generated by Image J software.

According to Equations (6) and (7), the magnification of the optical system is modulated by the displacement of the specimen, and the size and intensity of the specimen real image will also change with the displacement of the specimen, which will result in variation of the pixel value baseline. In this study, a bandpass filter is proposed to eliminate baseline drift. The implementation of the bandpass filter is based on fast Fourier transform (FFT) [35,36,37], and the calculation process is illustrated in Figure 5A. The originally captured image is firstly transformed from the spatial domain to the frequency domain via an FFT algorithm. Then a mask matrix or filter matrix is multiplied with the image in the frequency domain to eliminate or reduce the unwanted frequency component. Finally, the filtered image is transformed from the frequency domain back to the spatial domain by applying the inverse FFT algorithm. As the characteristics of the filtered image depend on the mask matrix, it is significant to choose a proper mask matrix or filter matrix. In this study, we utilized the built-in plugin in Image J software with the parameters shown in Figure 5B to generate the mask matrix, and the corresponding mask matrix is shown in Figure 5C. The axes of the mask matrix represent the frequencies in horizontal and vertical directions, respectively, with the zero-frequency component in the center. The mask matrix is a type of Gaussian bandpass filter and the pixel value of the mask matrix is the normalized magnitude, which ranges from 0 to 1. The white areas (pixel value = 1) will cause the corresponding frequencies of the original image to be filtered or removed completely, and the black areas (pixel value = 0) will cause the corresponding frequencies of the original image to be passed completely.

### 2.4. Method for Estimating the Position of Stripe

**Figure 6:**
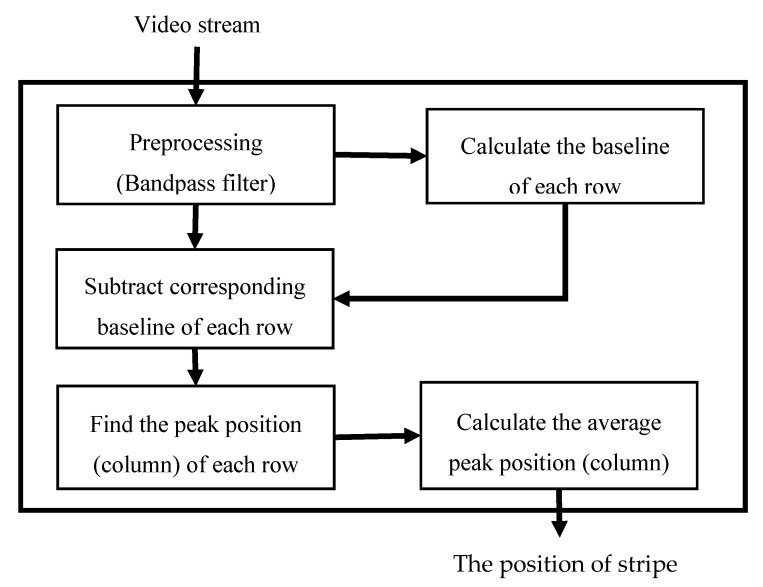
Flow chart for estimating the position of the stripe. In the algorithm, the input video frame is first preprocessed by a bandpass filter to eliminate baseline drift, and then the baseline of each row is calculated. Next, subtract the baseline of each row for the filtered image. Subsequently, find the peak value and corresponding position (column) for every row. Finally, calculate the average position (column) based on the peak position of individual rows.

In the present study, we use the average column coordinate of the stripe in the image coordinate system to represent the position of the stripe and propose a peak value detection method to estimate that position of the stripe. The estimation process is illustrated in Figure 6.

### 2.5. Image Acquisition and Processing

To improve the speed of image processing, the video stream was read by the CPU of a laptop (Inspiron 15-7559, Dell, Round Rock, US) from the USB interface and then immediately downloaded into a GPU (GeForce GTX 960M, NVIDA, Santa Clara, CA, US) processor for complex image processing. Finally, results were returned from the GPU back to the CPU after computation was completed. The code of the image acquisition and processing was written in C++ (Visual Studio, Microsoft, Redmond, WA, USA) with OpenCV and CUDA parallel computing library, respectively. The system acquired three color images (1280 × 720 pixel) at 30 frames/second. The images were displayed in real time on a computer monitor and streamed to the hard disk. The image acquisition and processing code could also display and record the average column of the stripe and the total time consumed in one sampling cycle in real time.

### 2.6. Functional Test Experiment

The purpose of the functional test experiment was to validate the experimental prototype shown in Figure 4 through a series of experiments. In functional test experiments, for simplicity, we used the screen of a cellphone to simulate the specimen, the experimental prototype was fixed in an optical platform and the cellphone was put on a 1-axis translation stage, which was placed underneath the objective of the prototype. The knob of the 1-axis translation stage was turned to simulate the movement of tissue. We first examined whether or not there was a stripe region in the capture image as theoretically predicted and whether the stripe region could move left and right as we moved the specimen up and down. Then, we analyzed the characteristics of the captured image in spatial and frequency domains. Finally, we validated the preprocessing method for baseline drift elimination.

### 2.7. Calibration Experiment

The purpose of the calibration experiment was to identify the mapping relationship between the displacement of living tissues in the world coordinate system and the movement of the stripe in the image coordinate system. In the calibration experiment, we used a piece of fresh heart tissue (pig) as the specimen, which was put on a dish and stained by a 0.01% solution of proflavine (Sigma-Aldrich, St. Louis, MO, USA) for imaging [28,29,30], and then put the dish on the 1-axis translation stage. To calibrate the prototype, the first step was to make the stripe appear in the right end through adjusting the distance between the specimen and the front end of the prototype, and the second step was to move the specimen up with a step of 0.5 mm by turning the knob of the translation stage. Then we repeated the second step until the stripe moved to the left end. Finally, we fitted the recorded experimental results via a linear function as shown below:

(9) y=ax+b

where x represents the displacement of translation stage, y represents the position (average column value) of the stripe in the image, and a,b were the parameters to be estimated by experimental results.

### 2.8. Animal Experiment

The purpose of the animal experiment was to examine the effectiveness and practicability of the prototype for measuring the movement of living tissue. In animal experiments, the prototype was mounted on a linear slider in the vertical direction, which could be moved up and down. Two SD rats were adopted, which were male, 8–10 weeks old, and around 220 g. The experimental rat was anesthetized by inhalational of isoflurane prior to preoperative skin preparation. Once the rat was fully anesthetized, it was mechanically ventilated with air supplemented with 2% isoflurane. Then, its abdomen was shaved using electric clippers and a small incision made (about 1 cm). The liver tissue was exposed by a mouth opener. Proflavine (0.01%) (Sigma-Aldrich, St. Louis, MO, USA) [28,29,30] was topically applied to the liver via a medical cotton swab before recording movement of the liver. To record the movement of the liver, we first adjusted the position of the prototype to make a stripe appear in the middle of the captured image. Then we started the data acquisition program to record the motion of liver tissue for 5 min. Finally, we plotted and analyzed the recorded results. The Animal Ethical and Welfare Committee (AEWC) at Nankai Hospital approved all animal studies (Approval No. NKYY-DWLL-2020-097).

### 2.9. Data Analysis and Processing

The experimental results were postprocessed and plotted using Origin software (Origin Lab, Northampton, MA, USA).

## 3. Results

### 3.1. Simulation

**Figure 7:**
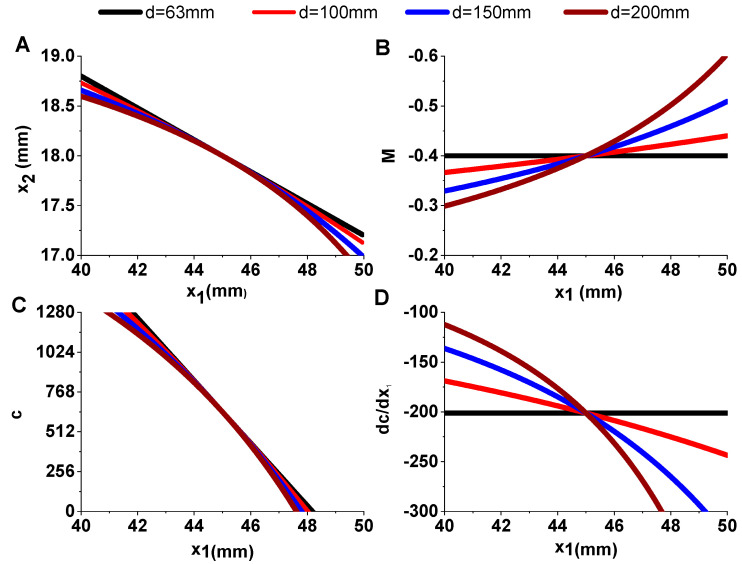
Simulation results with conditions of $d=63\;\mathrm{mm}\;\left(F_{1}+F_{2}\right)$, 100 mm, 150 mm, and 200 mm. (**A**) The changes of distance from image plane to optical lens II ($x_{2}$). (**B**) Magnification of optical system (M). (**C**) Position of stripe in the captured image (c). (**D**) Measurement sensitivity ($dc/dx_{1}$) with respect to distance from specimen to optical lens I ($x_{1}$).

According to the parameters provided by the website of Thorlabs, the effective focal lengths (EFL) of optical lens I and optical lens II are 45 mm and 18 mm, respectively. For the image sensor adopted in the prototype, the width (W) of the image area is 1.44 mm and the corresponding column number ($N_{c}$) is 1280. Substituting the above parameters into the derived Equations (6)–(8), and plotting the changes of distance from image plane to optical lens II ($x_{2}$), magnification of optical system (M), position of stripe in the captured image (c) with the changes of distance from specimen to optical lens I ($x_{1}$) in different d conditions, is shown in Figure 7.

Simulation results revealed that there is a perfect linear relationship between the movement of specimen and the movement of stripe in the captured image in condition of $d=F_{1}+F_{2}$ (black line in Figure 7A), and that the magnification of the overall optical system was unchanged (black line in Figure 7B). Nonlinear phenomenon will appear when the distance between the two optical lens is larger than the sum of their focal lengths, but it still can be approximately considered as linear (red line in Figure 7A) in condition of *d* = 100 mm. As nonlinear phenomenon increased gradually with deviation between d and $F_{1}$+$F_{2}\;$(blue and wine lines in Figure 7A), it was required that the distance of d to satisfy deviation ($d-F_{1}-F_{2}$) was as small as possible to avoid the severe nonlinear phenomenon.

Since the position of stripe (c) is represented by the corresponding column number of captured images and is in the range of 0 to 1280, the corresponding range of $x_{1}$ is 41.8~48.2 mm according to the black line in Figure 7C ($d=F_{1}+F_{2}$), which means that the theoretical maximum measurement range of the laboratory-built prototype is 6.4 mm and the theoretical resolution of the prototype is 6.4/1280 = 0.005 mm. According to Figure 7D, the theoretical sensitivity (derivative of c with respect to $x_{1}$) is −201/mm in an ideal situation.

### 3.2. Functional Test

**Figure 8:**
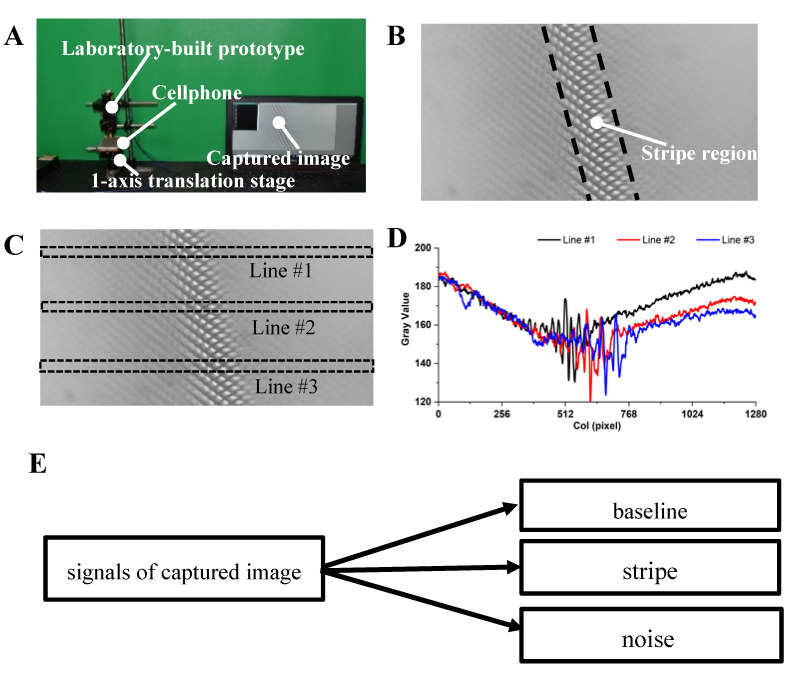
Functional test experiment. (**A**) Experiment setup including the laboratory-built prototype, a 1-axis translation stage and a cellphone. (**B**) Image captured by the laboratory-built prototype. (**C**) Dash line of the rectangular region is the selected region to be plotted. (**D**) The plotted profile of the selected region. (**E**) The captured image signal mainly consists of three components: baseline, stripe and noise.

In the functional test experiment, a cellphone was used as the specimen and put underneath the laboratory-built prototype, as shown in Figure 8A. The typical image captured by the prototype is shown in Figure 5B, from which we can see a clear stripe region. The pattern in the stripe regions was the microscopic image of the screen pixels array. The stripe regions in the captured image moved left and right as we moved the cellphone up and down.

To examine the characteristics of the pixel value in the stripe region and nonstripe region, we selected three different rectangle regions in the captured image, as shown in Figure 8C, and plotted the average pixel value with respect to column number, as shown in Figure 8D. It can be seen that the fluctuation amplitude of pixel value in the stripe region is greatly larger than that in the nonstripe region, which reveals that the position of the stripe can be estimated via detecting the fluctuation amplitude of the pixel value. The plotted profile in Figure 8D also reveals that the baseline of the pixel value is not constant, but varies from position to position, which makes it difficult to calculate the fluctuation amplitude around the baseline.

According to above analysis, it can be inferred that the captured image can be segregated into stripe component, baseline drift component and noise component, in which baseline drift signal and noise are the unwanted signals and should be eliminated or reduced as much as possible.

**Figure 9:**
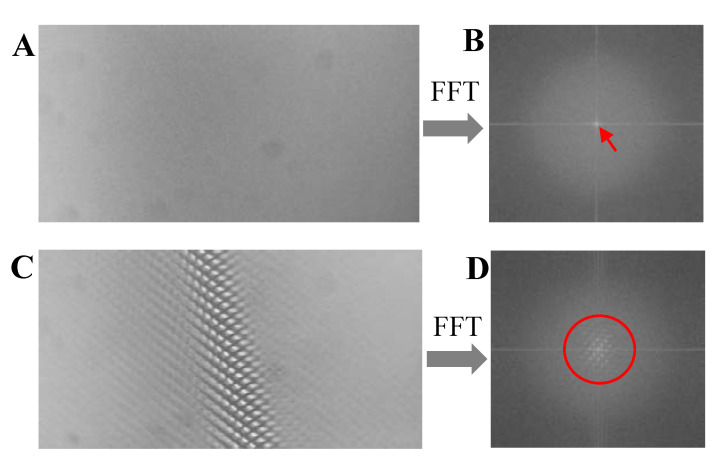
The frequency characteristic of the captured image. (**A**,**C**) Captured image in spatial domain without and with the presence of cellphone. (**B**,**D**) Captured image in frequency domain without and with the presence of cellphone.

Next, we transformed the captured image without (Figure 9A), and with (Figure 9C), the presence of the cellphone from the spatial domain to the frequency domain via FFT to analyze spectrum characteristics. The normalized power spectrum is shown in Figure 9B,D, respectively, in which the axes of the transformed image are the frequencies in horizonal and vertical directions, respectively, and the center point represents the DC component. The transformation results reveal that the baseline component is mainly in a circle area with a radius of 20 pixels (Figure 9B) and the stripe plus baseline component is mainly in a circle area with a radius of about 300 pixels (Figure 9D), which indicates that the stripe signal is mainly in a circle area with radius range from 20 to 300 pixels.

**Figure 10:**
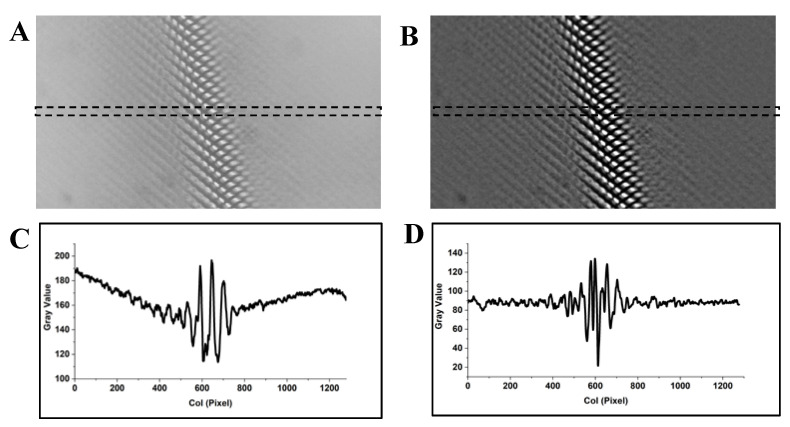
Comparison of the image in the spatial domain and the profile of the selected region before and after processing by bandpass filter. (**A**,**C**) Before processing by bandpass filter. (**B**,**D**) After processing by bandpass filter.

Finally, we examined the proposed image preprocessing method (a bandpass filter) to eliminate the effect of the baseline drift. An originally captured image and the corresponding filtered image are shown in Figure 10A,B, respectively. The profiles of the selected region of the original capture image and the filtered image are shown in Figure 10C,D, respectively. The comparison result reveals that the pixel value in the filtered image fluctuates around a horizon line (baseline) (Figure 10D), which indicates that the bandpass filter can perfectly eliminate the baseline drift. As the range of fluctuation is proportional to the clarity of the image, we can easily estimate the position (corresponding column number) of the stripe via detecting the fluctuation range around the baseline of the filtered image.

### 3.3. Calibration Experiment

**Figure 11:**
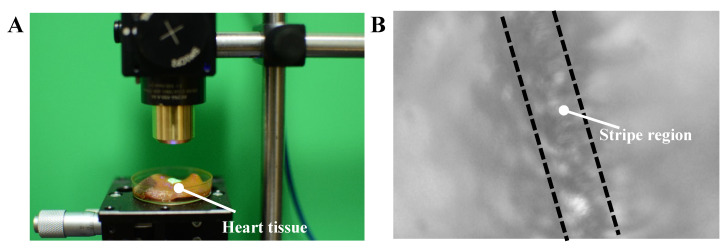
Calibration experiment. (**A**) A piece of heart tissue (pig) is placed in a dish and put on a 1-axis translation stage. Then the tissue is moved up by a step of length of 0.5 mm and the corresponding position of stripe is recorded in the image coordinate system. Above process repeated until the stripe moves from rightmost margin to the leftmost margin of the captured image. (**B**) Typical image of heart tissue recorded by the laboratory-built prototype during calibration experiment.

**Figure 12:**
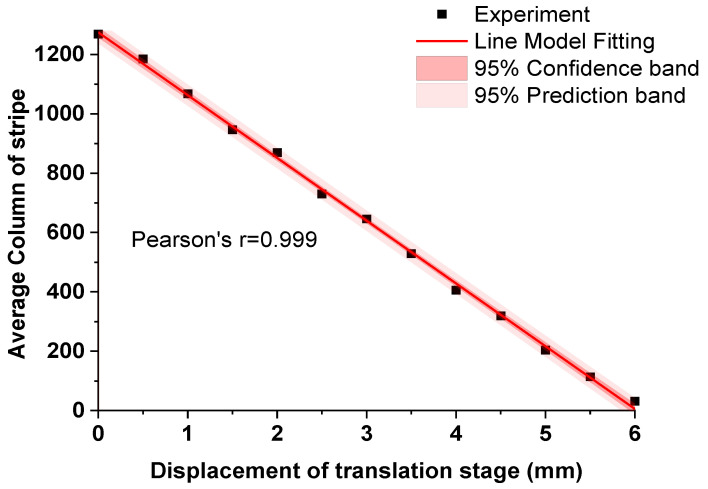
Calibration results of the laboratory built-prototype. Black squares represent the mean values of calculated stripe positions, red solid line is the fitting result, dark pink region represents 95% confidence band, and light pink region represents 95% prediction band.

The experimental setup and the typical image captured by the image sensor in the calibration experiment are shown in Figure 11A and Figure 11B respectively. The mapping relation between positions of the stripe (column number) in the recorded image and the displacements of the translation stage are shown in Figure 12.

The experimental calibration results (black squares) show that there is an almost linear relationship between the position of the stripe in the image coordinate system and the displacement of the translation stage in the world coordinate system. According to the fitted result, the slope and the intercept of the regressed linear equation are a=−211.46±2.21  mm^−1^ and b=1274.29±7.82, respectively. We also calculated the Pearson’s correlation coefficient between the fitting linear equation and experimental result, which is as high as −0.999. Calibration results reveal that the measurement range of the laboratory-built prototype is 6 mm, and the maximum indication error is less than 150 μm. The experimental results also reveal that it agrees well in measurement range and sensitivity ($\Delta c/\Delta x_{1}$) with theoretic estimated results.

### 3.4. Animal Experiment

**Figure 13:**
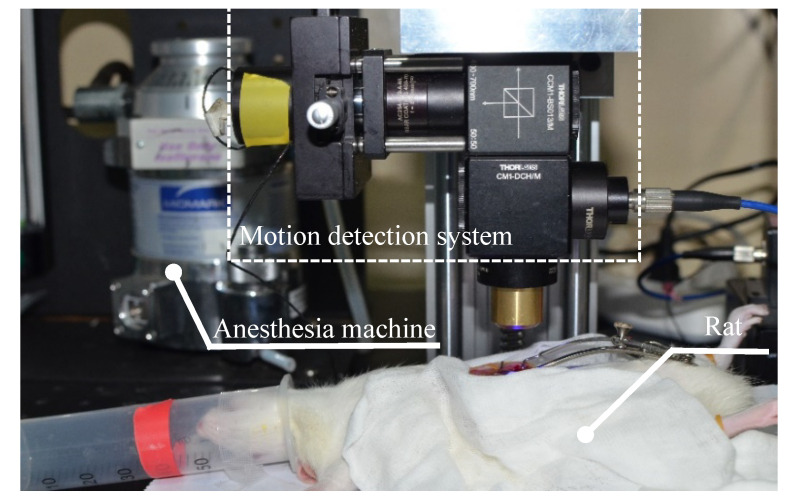
Photograph of setup for animal experiment. A rat was anesthetized and put underneath the laboratory-built prototype with its liver exposed. The period of recording the movement of liver was 5 min.

**Figure 14:**
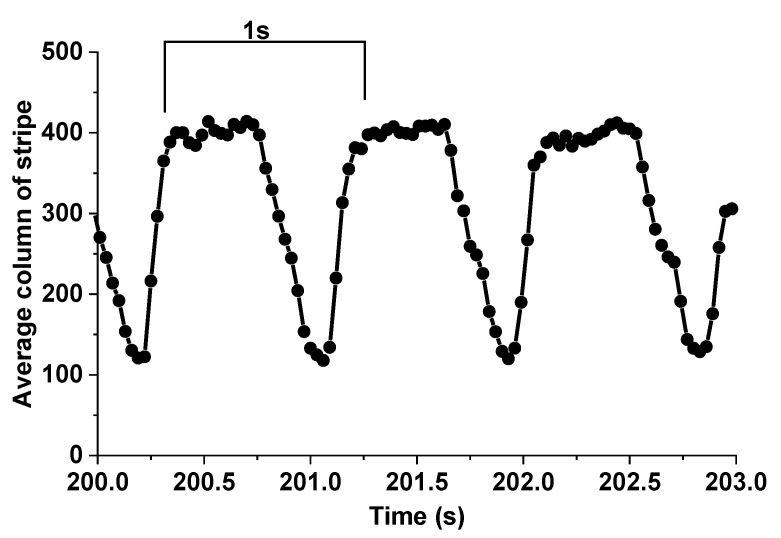
Motion curve for the liver of rat under anesthesia. The black dots represent the experimental results, while the solid line represents the predicted motion curve. The amplitude and frequency of the motion are roughly about 1.37 mm and 1 Hz respectively.

In order to demonstrate the effectiveness of the proposed method, we used the laboratory-built prototype to measure the movement of living rat tissues (liver) caused by respiration and heartbeat. Setup of the living animal experiment is shown in Figure 13, and the movement of liver in the image coordinate system is shown in Figure 14.

The experimental results in Figure 14 reveal that the frequency of the liver movement was roughly about 1 Hz, and the amplitude of the movement was about 290 pixels in the image coordinate system. According to the calibration results, the amplitude of the movement will be 1.37 mm in the world coordinate system. The movement at the liver of a living rat recoded by the prototype is very similar in amplitude, frequency and motion curve to the movement at the liver of a living mouse recorded by a laser displacement sensor [12,13], which has proved the effectiveness of our proposed method.

## 4. Discussions

In the present study, we proposed an image-based method to measure the motion of tissues caused by heartbeat and respiration, which has a compact structure and a high sampling rate to measure the motion of living tissues in vivo. We then established a laboratory-built prototype and validated the proposed method via simulation and experiments.

According to the Nyquist sampling theorem, in order to avoid distortion the sampling rate must be two times greater than the maximum frequency of the input signal [38]. In the proposed method, the sampling rate is dependent on the frame rate of the image sensor. In order to obtain the motion information of the tissue more accurately, it is necessary to make the frame rate of the sensor high enough to meet the sampling theorem. However, the sampling rate is not the bigger the better, since the signal-to-noise ratio (SNR) may decrease with the increasing sampling rate because of the decreased exposure time of the image sensor and the increased total effective noise [39,40]. Therefore, there is a trade-off between the sampling rate and the quality of the optical signals. In this study, animal experiment results reveal that the motion caused by respiration (1–2 Hz) can be recorded by the prototype under a frame rate of 30 fps, but the motion caused by the heartbeat cannot be recorded because the heart rate of rat is higher (5–10 Hz) than respiration. For humans, the heart rate is roughly about 1–2 Hz [41,42] (similar to the respiration rate of the rat), and the respiration rate of humans is significantly lower compared with heart rate, which is about 0.2 Hz [43]. Moreover, previous studies have shown that the motion caused by heartbeat is ∼100 times smaller than the displacement induced by respiration, except in the heart tissue itself [12,13]. Thus, we can infer that the frame rate of 30 Hz (video frame) is enough to measure the motion of human tissues caused by heartbeat and respiration.

As the major goal of the proposed method is for the integration into a minimally invasive instrument (diameter: ~10 mm) [44,45,46], a simple structure is emphasized in this study. In the proposed method, the optical system is almost the same as a wide-field fluorescence microscopy system, which only contains several simple optical elements and an image sensor placed in a straight line. The size of the proposed optical system depends on the diameter of the optical lens and the size of the image sensor. Although the laboratory-built prototype is too big to be integrated into a minimally invasive instrument, the method for measuring the motion of tissue was validated by the prototype. Since the image sensor adopted in the prototype is small enough (2.53 mm × 1.53 mm) to be integrated into a minimally invasive instrument, we can replace the standard objectives by optical lenses with a small diameter to realize miniaturization.

One of the limitations in this study is that we use the fluorescent agent of proflavine to enhance the intensity of the fluorescence signal which, however, increases the complexity of operation in minimally invasive surgery. Moreover, the exogenous fluorescent agent may have influence on the normal physiological activities of living animals. Compared with an exogenous fluorescent agent, an endogenous fluorescent molecule (e.g., NADH, FAD) seems to be a better option to measure the motion of tissues [47,48,49]. Thus, the above limitations can be overcome by using an image sensor with high speed and high quantum efficiency (e.g., Electron Multiplying CCD) and utilizing autofluorescence for imaging, but it still needs to be validated by experiments in the future.

## 5. Conclusions

In the present study, we proposed an image-based method to measure the longitudinal motion of tissues, which has a simple structure and is easy to integrate with a minimally invasive instrument (~10 mm). To validate the proposed method, we developed a laboratory-built prototype and performed a series of functional test experiments. We also built a mathematical model of the prototype using an ABCD matrix method and analyzed parameters such as measurement range and sensitivity. Then, we calibrated the prototype using a piece of heart tissue (pig). Calibration results revealed that the measurement range was 6 mm, the sensitivity about 211.46 mm^−1^ and the maximum indication error less than 150 μm, which is close to the corresponding model analysis results. Finally, we utilized the prototype to measure the tissue motion of a living rat under anesthesia in vivo. Animal experiment results revealed that the motion curve was similar with a previously published motion curve recorded by a laser displacement sensor.
